# Low-Cost Carbon Fibre Derived from Sustainable Coal Tar Pitch and Polyacrylonitrile: Fabrication and Characterisation

**DOI:** 10.3390/ma12081281

**Published:** 2019-04-18

**Authors:** Omid Zabihi, Sajjad Shafei, Seyed Mousa Fakhrhoseini, Mojtaba Ahmadi, Hossein Ajdari Nazarloo, Rohan Stanger, Quang Anh Tran, John Lucas, Terry Wall, Minoo Naebe

**Affiliations:** 1 Institute for Frontier Materials, Deakin University, Geelong, VIC 3216, Australia; omid.zabihi@deakin.edu.au (O.Z.); s.shafei@deakin.edu.au (S.S.); sfakhrho@deakin.edu.au (S.M.F.); ahmadim@deakin.edu.au (M.A.); hossein.nazarloo@deakin.edu.au (H.A.N.); 2 Department of Chemical Engineering, The University of Newcastle, Callaghan, NSW 2308, Australia; rohan.stanger@newcstle.edu.au (R.S.); quanganh.tran@newcstle.edu.au (Q.A.T.); John.Lucas@newcstle.edu.au (J.L.); Terry.Wall@newcstle.edu.au (T.W.); 3 School of Engineering, Edith Cowan University, 270 Joondalup Drive, Joondalup, Perth, WA 6027, Australia

**Keywords:** low-cost carbon fibre, polyacrylonitrile, coal tar pitch, electrospinning

## Abstract

Preparation of high-value pitch-based carbon fibres (CFs) from mesophase pitch precursor is of great importance towards low-cost CFs. Herein, we developed a method to reduce the cost of CFs precursor through incorporating high loading of coal tar pitch (CTP) into polyacrylonitrile (PAN) polymer solution. The CTP with a loading of 25% and 50% was blended with PAN and their spinnability was examined by electrospinning process. The effect of CTP on thermal stabilization and carbonisation of PAN fibres was investigated by thermal analyses methods. Moreover, electrospun PAN/CTP fibres were carbonised at two different temperatures i.e., 850 °C and 1200 °C and their crystallographic structures of resulting such low-cost PAN/CTP CFs were studied through X-ray diffraction (XRD) and Raman analyses. Compared to pure PAN CFs, the electrical resistivity of PAN/25% CTP CFs significantly decreased by 92%, reaching 1.6 kΩ/sq. The overall results showed that PAN precursor containing 25% CTP resulted in balanced properties in terms of spinnability, thermal and structural properties. It is believed that CTP has a great potential to be used as an additive for PAN precursor and will pave the way for cost-reduced and high-performance CFs.

## 1. Introduction

Due to outstanding specific strength and modulus, carbon fibres (CFs) have been used commercially in structural composite applications for high performance, light-weighting as well as ergonomics [1,2,3,4]. The global market for carbon fibre is anticipated to triple in one decade and exceed 100 thousand tonnes in 2020 with a value of over $3 billion [5]. While carbon fibre composites are currently used in aircraft and some racing cars, the cost of CFs is prohibitively high for many other industry sectors. The high price of CFs is directly related to the cost and yield of the precursor from which it is obtained and the cost of its conversion. The vast majority of commercial CFs are currently produced from polyacrylonitrile (PAN) which is an expensive precursor, contributing to ~51% of the price of carbon fibre.

Since there is an increasing demand for commercialisation of CFs in various industries, development of low-cost CFs is under extensive investigations. Given the significant contribution of the precursor to the final price of CFs, an investigation into alternative precursors can potentially lead to materials and process developments that can result in the development of low-cost CFs [6]. The most important alternatives for CF precursors are pitch [7,8] and lignin [9,10,11]. Although lignin as a renewable precursor has potential to significantly reduce the cost of carbon fibre, there are still challenges in terms of commercialisation due to the low mechanical performance of lignin-based carbon fibres [12]. Pitch is derived from the remnants of crude oil or coal distillation that are rich in aromatic hydrocarbons [13,14]. While pitch-based CFs can be manufactured with higher tensile modulus, the tensile strength of pitch-based CFs is often lower than that of PAN-based CFs [15]. Pitch-based CFs are more suitable for applications such as aircraft brakes and space satellite components, where heat dissipation and electrical conductivity are more critical. 

Pitch is composed of aromatic compounds, with 200–300 g/mol average molecular weight, which can be increased to 1000 g/mol by a solvent extraction process. Pitch-based CFs are usually obtained from coal tar or petroleum residues [3]. A relatively high price of the petroleum pitch-based precursor, as well as the depletion of their sources, is the main reason to incline the use of coal-tar pitch instead of petroleum pitch. Coal-tar pitch, which is a by-product of the coke oven, is frequently used as a binder and impregnator in the manufacture of electrodes in the aluminium and steel industries. Coal-tar pitch can be also used as a matrix precursor for carbon/carbon composite due to high carbon yield. However, the use of coal tar as CFs precursor requires physico-chemical treatment for extraction and purification of its pitch [3]. 

Extensive research on electrospinning has been conducted because non-woven web consisting of polymeric fibres with diameter down to hundreds of nanometres can be produced through electrospinning process [16,17,18,19]. In this regard, the composition of PAN with a petroleum-based pitch for fabrication of CF precursors has been widely investigated. Liu et al. [15] discussed that the thermodynamic miscibility of PAN with petroleum-based pitch is the main challenge in electrospinning of the PAN/pitch fibres. Park et al. [14,20] have successfully prepared non-brittle carbon fibres by the electrospinning of pitch extracted from pyrolyzed fuel oil. In another study by Bui et al. [17] electrospinnability of the petroleum-based pitch was investigated using PAN blends. Recently, Yang et al. [21] extracted coal-derived material from residues of the hyper-coal process in order to refine its pitch to be melt-spinnable for carbon fibre manufacturing. In a recent study by Stanger et al. [22], Australian sourced whole coal samples were employed as the carbon fibre precursor and a customised thermal extrusion for direct melt spinning of coal with high fluidity coking properties was used for direct CFs production. However, the thermally extruded CFs were not spun down to commercial CF diameters. Moreover, the resulting extruded CFs were relatively porous having microvoids which in turn resulted in low mechanical performance. In this work, we blended coal-tar pitch (CTP) and PAN in organic solvents and evaluated the spinnability of the blends using electrospinning. Preliminary evaluation of formed fibres using the electrospinning process can provide useful information on the spinnability of the blends in different ratios. Inspired by the high molecular weight of extracted CTP, we hypothesised that electro spinnable solutions can be prepared by varying the solution concentration and ratio of CTP to PAN, which can be processed into low-cost CFs. To verify this hypothesis, electro-spinnability of a PAN solution containing high loadings of CTP along with its rheology behaviour was examined and various properties of the precursor and the final stabilised/carbonised CFs were investigated. Such electrospun carbon fibres can be potentially used in applications such as batteries [23,24], electrodes of capacitors and supercapacitors [25,26], filters [27] and catalysts [28]. Electrical, thermal and morphological properties of the fibre mats were studied to evaluate the suitability of the membranes for these applications. 

## 2. Materials and Methods

### 2.1. Materials

Polyacrylonitrile (PAN) with a molecular weight of 85 kg/mol was purchased from Goodfellow. Dimethylformamide (DMF) and Tetrahydrofuran (THF) were purchased from Sigma-Aldrich (Sydney, Australia). The coal tar pitch was derived from a Queensland coking coal whose properties are given elsewhere [9].

### 2.2. Extraction of CTP

**Figure 1:**
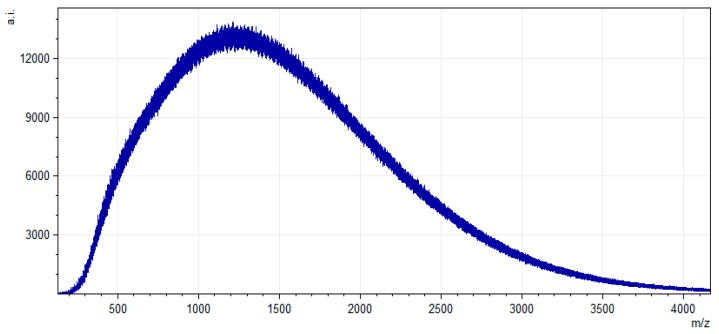
Molecular weight distribution of combined CTP material using laser desorption/ionization-time of flight mass spectroscopy (LDI-TOF-MS). The spectrum was acquired using a total of 2000 shots at 90% laser power.

The coal tar pitch material was produced by heat-treating coking coal to 400 °C, quenching the semi-coke and applying solvent extraction. These “low-temperature pitches” are expected to contain more reactive structures than those from a coke oven which are exposed to high temperatures in the coking process. Approximately 200 g coal was heated in four batches of 50 g at 5 °C/min to 400 °C. This pre-treatment temperature was selected based on previous work with this coal that corresponds to the point of initial thermal extrusion [22] (demonstrating sufficient thermoplasticity). Other work in this field has shown that solvent extracts from semi-cokes produced after softening contain a higher molecular weight distribution. The semi-coke samples then underwent solvent extraction in two 100 g batches. A solvent extraction wash was carried out with 100 g of sample in 500 mL of THF. The resulting solution was then placed in an ultrasonic bath for 3 h at 40 °C. The solid residue was filtered out with a vacuum filter and the extract containing CTP was collected by evaporating off the THF. This process was performed twice for each 100 g batch of semi-coke samples then combined to obtain 13 g of extracted CTP. The extraction yield was found to be approximately 6.5% with respect to the raw coal. Figure 1 shows the molecular weight distribution of the combined coal tar pitch extract, determined by laser desorption/ionization-time of flight mass spectroscopy (LDI-TOF-MS) using methods from previous work [22]. The CTP may be summarised as having an average MW of 1375 Da with 90% of the material under 2316 Da (i.e., MW 90).

### 2.3. Preparation of Electrospinning Solutions

Solutions of U0, U2 and U4 were prepared for electrospinning with CTP to PAN mass ratio of 0%, 25% and 50% respectively. To do so, first solid particles of CTP and PAN powder were added together with the above ratios. The ratio of THF to DMF was maintained at the mass ratio of CTP/5 to PAN in each solution. To have a spinnable solution, THF and DMF were added into the solutions in a way that mass of CTP/5 + mass of PAN had a total concentration of 12 wt % according to the following equation.

(1) $$\frac{\frac{\mathrm{CTP}\;\mathrm{Mass}}{5}+\mathrm{PAN}\;\mathrm{Mass}}{\frac{\mathrm{CTP}\;\mathrm{Mass}}{5}+\mathrm{PAN}\;\mathrm{Mass}+\left(\mathrm{THF}-\mathrm{DMF}\right)\;\mathrm{Mass}}=12\%.$$

### 2.4. Electrospinning of the Solutions

Fibres were collected on a rotating 12 cm diameter drum at the speed of 1.4 krpm placed 15 cm away from the tip of the needle (Gauge 21G). Solutions were fed at the feed rate of 1 mL/hr and a voltage of 18–22 kV was applied between the needle and the collector. 

### 2.5. Stabilisation and Carbonisation of the Fibres

The electro-spun fibre mats with dimensions of 12 × 5 cm^2^ were stabilised in an air-convection oven in four steps, each step taking 20 min in the range of 232–258 °C. Then, stabilised fibre mats were carbonised by heating in a tube furnace with a heating rate of 10 °C/min to reach 850 and 1200 °C, holding for 1 min. Both stabilisation and carbonisation processes were performed under tension by fixing the mats within a metal clamp and applying a ~30 g load. 

### 2.6. Characterisations

A rheometer (Discovery HR-3, TA Instruments, New Castle, DE, US). was used to measure the viscosity of the solutions as a function of shear rate ranging from 0.1 to 100 s^−1^ (40 mm diameter geometry, 2° cone angle in 49 µm truncation gap) at room temperature. Scanning electron microscopy (SEM) images were taken from the surface of the fibres using Zeiss Supra (Mönchhofallee, Kelsterbach, Germany).55VP at a 5 kV electron voltage. The fibre diameter of electro-spun fibres was measured using SEM images through image processing with Image J software and 100 measurements were conducted. Thermogravimetric analysis (TGA) measurements were obtained by a Perkin–Elmer TGA instrument (Waltham, MA, USA). at the heating rate of 10 °C/min under nitrogen atmosphere with a flow of 60 mL/min. Differential scanning calorimetry (DSC) analyses were conducted by a TA Q200 DSC instrument C at various heating rates under an air atmosphere. Brunauer, Emmett and Teller (BET) surface area was measured by a Micromeritics TriStar 3000 (Norcross, GA, USA) sing adsorption isotherm of nitrogen at 77 K. The X-ray diffraction (XRD) measurements were performed by a PANalytical X'Pert Pro (Jarman Way, Royston, UK) Diffractometer (Cu Kα radiation with λ = 1.54184 Å), operating at 40 kV and 30 mA with a step size of 0.033. Size of PAN and graphite crystallites in the pristine and carbonised electro-spun mat was calculated using the Scherrer equation according to the following:

(2) $$\tau =\frac{k\lambda }{\beta \cos\theta },$$

where “*k*” is the shape factor of crystallites (0.9), "*β*" is full width at half maximum (FWHM) of the crystalline peak at a diffraction angle of "*θ*” and “*τ*" is the mean crystallite size. To calculate FWHM, a multiple peak fitting approach will be applied on XRD curves using Origin software to eliminate the effect of background inclination on the accuracy of results.

The CF samples were analysed with Renishaw inVia Raman microscope and formation of disordered sp3 and graphitic sp^2^ carbon atoms (D- and G- bands, respectively) was investigated. Four random spots on each sample were picked to measure ID/IG ratios of samples. A conductivity meter (S30 Mettler Toledo SevenEasy) was used to measure the conductivity of the solutions. The electrical sheet resistance of the fibre mats was measured in fibre direction in 10 spots using a SZT-2A four-point probe tester. The molecular weight distribution of the CTP was determined using a Bruker (Billerica, MA, USA) Daltonics UltrafleXtreme MALDI TOF/TOF. The LDI-TOF-MS mass profile of the extract was then acquired using a smartbeam II laser (Nd:YAG, 355 nm) in positive, reflectron mode.

## 3. Results

### 3.1. Spinnability

**Figure 2:**
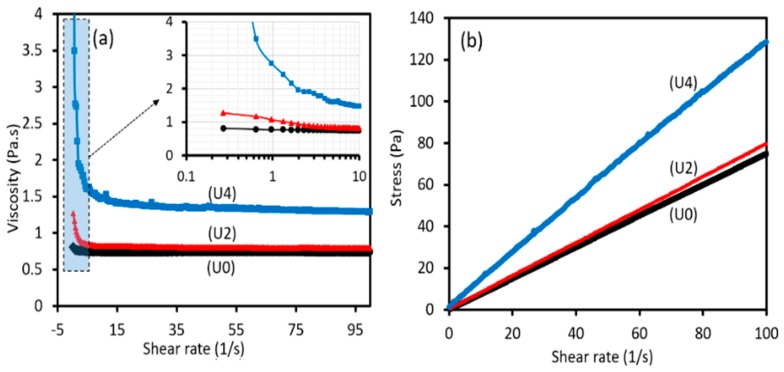
Viscosity-shear rate curves (**a**), and stress-shear rate curves (**b**) for various coal tar pitch/ polyacrylonitrile (CTP/PAN) solutions.

**Figure 3:**
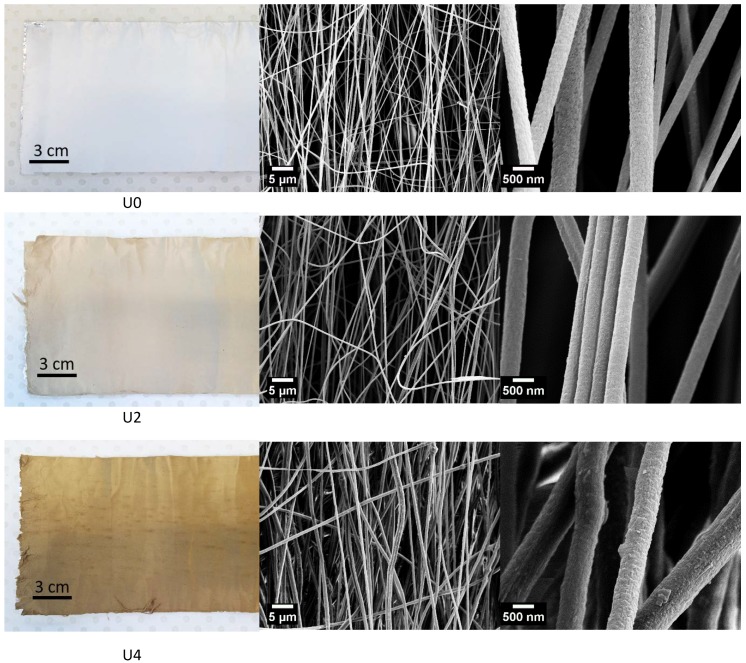
Left column: optical images of fibre samples, middle column: scanning electron microscopy (SEM) images of samples at low magnification and right column: SEM images of samples at high magnification for 0% (U0), 25% (U2) and 50% (U4) CTP in rows 1, 2 and 3, respectively. As the amount of CTP in PAN increases, the colour of the fibre mats turn to gold-brown and the stack of fibres became thicker.

When it comes to processing and property relationships in electrospinning, different processing and materials factors including solution concentration, spinning atmosphere effects, accelerating voltage, distance from tip to target and solution viscosity play pivotal roles in determining the final properties of electrospun fibres. The viscosity of solutions can affect initiating droplet shape, jet trajectory, and fibre diameter. Moreover, the increase in fibre diameter can arise from the increment of the viscosity [29,30]. Based on this discussion, the effect of viscosity on the morphological aspects of spun fibres was studied. With respect to viscosity, the change of shear stress and viscosity according to shear rate are shown in Figure 2a,b. Moreover, the morphology of the electrospun fibre mats was studied using SEM analyses as shown in Figure 3. Tukey’s ANOVA analysis shows that statistically significant increases of the fibre diameter were noted by the increase in the amount of CTP from 0% to 50%. Moreover, as presented in Table 1, U2 sample possesses a lower standard deviation compared to the U4 sample. In other words, more uniformity in fibres containing 25% CTP was observed and incorporation of 50% CTP reduces the uniformity of the produced fibres. As can be seen from Figure 2a, the viscosity of all three solutions decreased with an increase of the shear rate, which denotes that PAN/CTP solutions behave as non-Newtonian fluids. For non-Newtonian fluids, various mathematical models can be used to fit the relationship between shear stress and shear rate. Among them, the Power law model is the most commonly used method to describe this behaviour according to the following equation [31,32]:

*Ƭ* = *K* × *ϒ*^n^, (3)

where *Ƭ* is the shear stress, *K* is the flow consistency coefficient, *ϒ* is the shear rate, and *n* is the flow behaviour index. With the power law model, the flow consistency coefficient and flow behaviour index can be obtained. Flow behaviour index indicates whether the solution is Newtonian or non-Newtonian. Power law model parameters were extracted which are presented in Table 1. As shown, the addition of 25% CTP to PAN solution (U2 sample) increases the viscosity. Such increment in viscosity can lead to an increase in fibre diameter, and negatively affect the spinnability, as seen in Figure 3. A similar trend can be also seen for PAN solution containing 50% CTP (U4 sample). Additionally, the shear thinning phenomena can be detected in viscosity behaviour of both PAN solutions containing 25% and 50% CTP. To study the shear thinning properties, flow behaviour index (*n*) obtained from the Power law equation is reliable. Accordingly, the decreases of n from 0.99 for pure PAN solution to 0.95 and 0.92, respectively for U2 and U4 samples, can be evident for such deduction. 

The presence of CTP in PAN solution, on one hand, can result in local interactions between these two ingredients (e.g., dipole-dipole interactions); therefore, the solution shows initial resistance towards shear rate. On the other hand, in the presence of CTP, the PAN polymer chains can be aligned easier, which in turn can lead to shear thinning behaviour. The reason behind this can possibly be attributed to the increases in the solution inertia [33]. Generally, it is hypothesised that the addition of CTP to PAN solution can result in an adverse effect on the electrospinning process. Moreover, the inclusion of CTP can lead to the drop in charge density of PAN solution and consequently decrease in repulsion forces, as evidenced by a decrease in solution electrical conductivity. This effect can be detrimental to achieving dense and smooth fibres [34]. To investigate the potential of fibre to carry the charges, electrical conductivity measurement was performed. As shown in Table 1, compared to U0, the reduction in electrical conductivity of the U4 sample doubled compared to the U2 sample. Therefore, as discussed above, the non-uniformity of fibres is much more profound for U4 sample, compared to U2 sample. This non-uniformity can be evaluated by comparing the standard deviation of calculated average diameters for different samples, denoting diameter distribution for U4 sample is significantly non-uniform. On the other hand, generally, the reduction of the n value is considered as the indication of PAN chains entanglement, which is a vital aspect for fibres deposition during the electrospinning process [35]. Therefore, although the increase in fibre diameter is observed (U2 sample), the decrease of standard deviation in the diameter of U2 sample suggests the electrospun nanofibers have uniform fibre diameters, compared to the U0 sample.

### 3.2. Thermal Analyses

**Figure 4:**
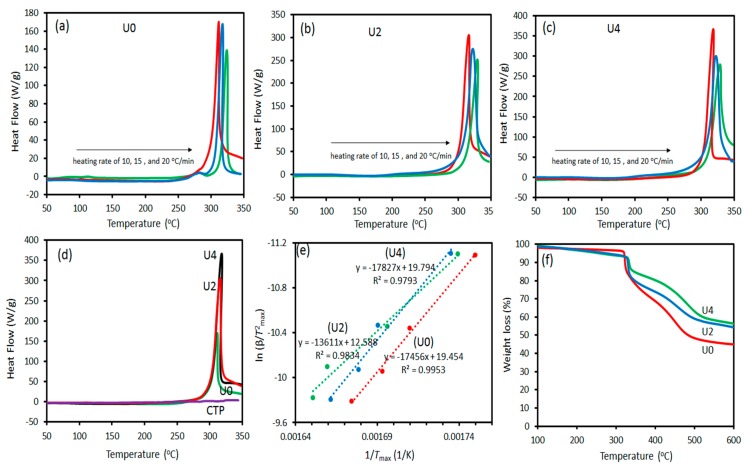
Differential scanning calorimetry (DSC) thermograms at various heating rates for (**a**) U0, (**b**) U2 and (**c**) U4, respectively; noting the change in scale of the Y-axis. Comparison of DSC thermograms at a heating rate of 10 °C/min (**d**), Kissinger plots for obtaining the activation energy of thermal stabilisation process (**e**), and TGA thermograms of various samples (**f**).

The effect of CTP on thermal conversion (i.e., stabilisation and carbonisation) of the PAN polymer to its corresponding CFs, was investigated by DSC and TGA thermal analyses. In this regard, DSC analyses of various PAN/CTP fibres in air atmosphere can mimic the stabilisation process while the carbonisation process can be simulated by TGA analyses in an inert atmosphere. Figure 4a–c shows DSC thermograms of various samples at three different heating rates, in which all thermal stabilisation temperatures (*T*_s_) were shifted to higher temperatures while the heat of reaction (Δ*H*_s_) was decreased by an increase in the heating rate. For better comparison, the thermal stabilisation characteristics of various samples obtained by DSC are shown in Figure 4d and Table 2. As shown, at a constant heating rate e.g., 10 °C/min, Δ*H*_s_ was significantly increased by increasing CTP content. For example, Δ*H*_s_ of the U2 and U4 samples was two times higher than U0 as a result of CTP presence. This is while DSC thermogram of pure CTP does not show any heat trace in the given temperature range (>350 °C). Moreover, the addition of CTP to PAN fibre does not change *T*_s_ significantly. It is worth mentioning that lower *T_s_* and higher Δ*H*_s_ are highly desirable for a more efficient thermal stabilisation process. In other words, addition of CTP not only won’t postpone the stabilisation process temperature but also facilitates the stabilisation process through increased heat release during the process, which overall means a lower energy consumption in stabilisation process of fibres. This discussion can also be supported by obtaining the activation energy required for thermal conversion (*E*_s_) during the stabilisation process. Here, we used the Kissinger method to evaluate *E*_s_ according to the following equation [36,37]:

(4) $$ln\;\left(\frac{heating\;rate}{T_{s}^{2}}\right)=\frac{-E_{s}}{RT_{s}}+constant,$$

where R is the gas constant. Taking the *T*_s_ for each heating rate into Equation (4), Figure 3e depicts a logarithm plot for the reciprocal of the absolute *T*_s_ versus the value of heating rate/$T_{s}^{2}$. The obtained values of *E*_s_ for three samples are presented in Table 2, which show that *E*_s_ decreased for the U2 sample compared to the U0 sample while a negligible increase was observed for the *E*_s_ value of U4. TGA analyses in an inert atmosphere can provide an insight into fibres behaviour in the carbonisation process. Two parameters in PAN fibres containing CTP were evaluated by TGA analyses. Firstly, the temperature in which the carbonisation process begins to occur (*T*_c_) and secondly, the remained carbon content at high temperatures which shows the elimination of oxygen and nitrogen atoms from a fibre structure. Moreover, a high carbon yield at high temperatures may also denote a high carbon content in the original precursor. Generally, a high carbon yield of CFs is of importance in terms of both mechanical and electrical performance. Figure 4f shows TGA thermograms of various samples and their results are presented in Table 2. As shown, although *T*_c_ shifted slightly to higher temperatures upon the addition of CTP, the carbon yield at 600 °C significantly increased from 43% for pure PAN fibre to 54% and 56% for PAN fibres containing 25% and 50% CTP, respectively. This increase in carbon yield is a result of the addition of CTP, which is a distribution of macromolecules containing high aromatic carbon content.

### 3.3. Crystallographic Structures

**Figure 5:**
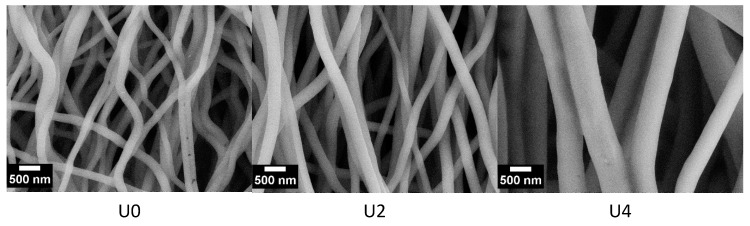
SEM images of the CFs carbonised at 850 °C containing 0%, 25% and 50% CTP. Average diameter (Mean ± SD) for the carbonised samples U0, U2 and U4 was 0.25 ± 0.06, 0.28 ± 0.07 and 0.57 ± 0.15 respectively.

Electrospun fibres were stabilised and carbonised prior to XRD and Raman studies. SEM images of the CFs produced from samples U0, U2 and U4 are shown in Figure 5. During stabilisation and carbonisation, fibres underwent a shrinkage, resulting in thinner fibres with smoother surface compared to precursor fibres. 

**Figure 6:**
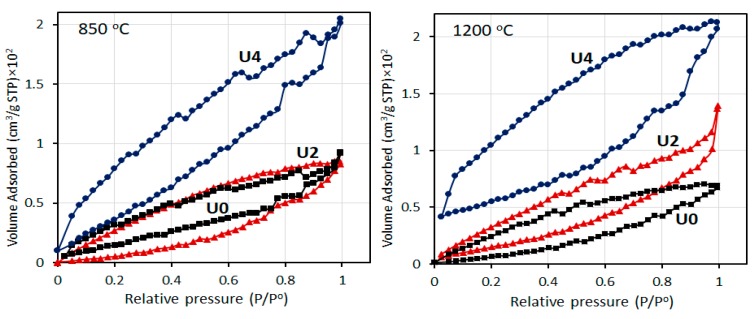
Isothermal adsorption/desorption of nitrogen in various CFs.

Figure 6 shows the adsorption/desorption isotherms of nitrogen at 77 K for various CFs samples. All the isotherms showed the typical type III representing pore adsorption. Based on these results, specific surface areas and pore size are obtained and presented in Table 3. It can be seen that specific surface area and pore size increase with increasing CTP content in both carbonisation temperatures of 850 °C and 1200 °C, which is probably due to porous structure and microvoids in CTP. Increasing carbonisation temperature also results in a higher surface area. However, in U4 sample, an increase of carbonisation temperature from 850 °C to 1200 °C did not change the surface area significantly.

**Figure 7:**
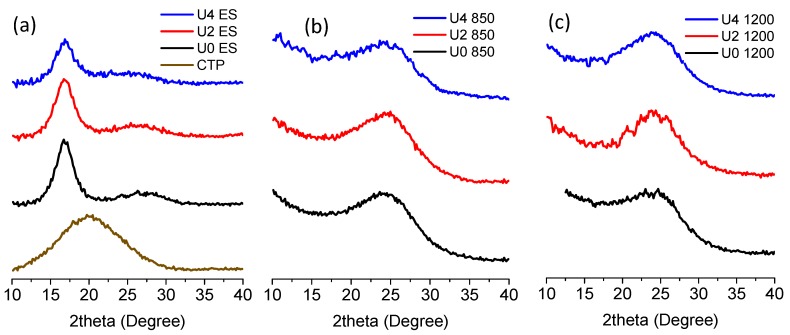
X-ray diffraction (XRD) patterns of precursors (**a**), and carbonised fibre at 850 °C (**b**) and 850 °C (**c**) for U0, U2 and U4 samples.

As discussed earlier, the addition of CTP as a miscible dope additive to PAN/DMF spinning dope reduces the strong binary interaction force between PAN polymer chains, which causes an increase in the crystallisation degree of fibres. Subsequently, the larger size of precursor crystallites will result in more developed cyclised PAN polymer and the larger graphitic structure in carbonised fibres [38]. Figure 7a shows XRD patterns of CTP and control fibre (U0 sample). As expected for the CTP sample, a broad amorphous peak at 2θ = 20.15° was observed and considered for further quantifications. Adding 25 and 50% CTP to PAN shifted the amorphous peak of PAN from ~25.5° to ~23.5°, which is because of convolution of 20.15° and 25.5° peaks.

As presented in Table 4, calculated size of (1 0 0) crystallographic plane for U0, U2 and U4 samples showed an increase in the size of the (1 0 0) crystalline plane from 2.59 nm in the U0 sample to 2.63 nm and 2.74 nm in the U2 and U4 precursor samples, respectively. As all samples were prepared under the same experimental conditions, a slight growth in the size of the crystallites is described by adding CTP as the dope additive. Adding CTP as a dope additive decreases the strong dipole-dipole interaction between cyanide groups in PAN chains. Therefore, the presence of CTP reduces the strong cohesive bonding between PAN chains which causes the formation of PAN clusters in the spinning dope. The higher CTP load in the spinning dope resulted in the lower polymer chains interaction and this resulted in the formation of larger PAN crystallites in the electrospun fibres, as presented in Table 3. The reduction of interaction in PAN chains due to the addition of CTP was also confirmed by observation of the shear-thinning behaviour in PAN/CTP solutions. Low-temperature carbonisation of stabilised fibres at 850 °C did not reveal a distinguishable difference between samples as shown in Figure 7b. Further calculation and quantification of low-temperature carbonised samples revealed that addition of both 25% and 50% CTP cannot change the size of the (0 0 2) crystallographic planes and it remained at 1.07 nm. However, the crystallographic structure of three samples evolved differently after carbonisation at 1200 °C as shown in Table 4. High-temperature carbonisation did not improve the size of the (0 0 2) crystalline plane at the U0 and U2 samples compared to low-temperature carbonisation. This is while the size for the U4 sample decreased to 1.04 nm at 1200 °C, the resultant reduction in the size of the crystallite in the U4 sample shows the better integrity between CTP and PAN at a lower amount of CTP e.g., 25% in the spinning dope.

**Figure 8:**
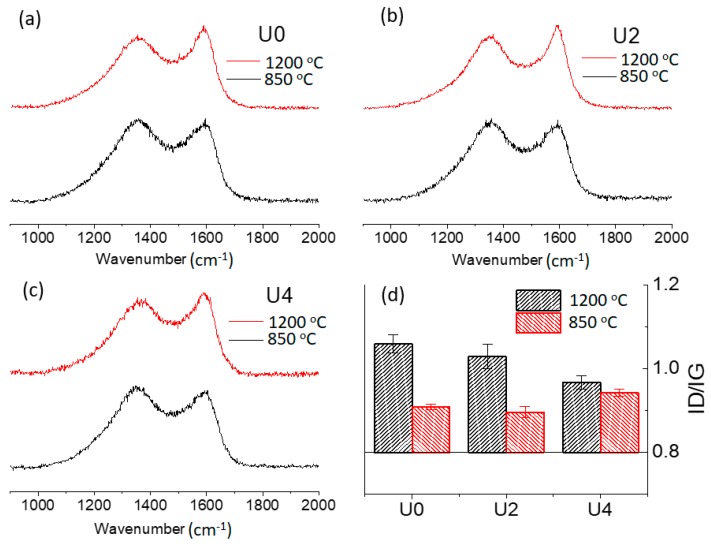
Raman spectra and D/G area ratios for carbonised PAN and CTP/PAN samples at different carbonisation temperatures (**a**–**c**); I_D_/I_G_ ratios are the average of four different measurements (**d**).

Raman spectroscopy was also carried out to investigate the influence of inclusion of CTP as an additive to the PAN spinning dope. As shown in Figure 8, a notable reduction in D-band (defect structure) was observed after increasing carbonisation temperature from 850 °C to 1200 °C. Higher intensity of G-band (graphitic structure) at 1200°C is attributed to the developing sp^2^ carbon atoms at a higher temperature, which demonstrates higher graphitic like carbon atoms. 

Low-temperature carbonisation of U0, U2 and U4 samples at 850 °C resulted in 1.06, 1.03 and 0.97 I_D_/I_G_ ratios, while carbonisation at 1200 °C reduced I_D_/I_G_ ratios to 0.91, 0.90 and 0.94, respectively. The lower I_D_/I_G_ ratios denote that better graphitic structures were formed and CTP was integrated to PAN graphitic structure more efficiently. The lowest I_D_/I_G_ ratios belonged to U4 at low carbonisation temperature and U2 at a high carbonisation temperature. This can be due to the thermal condition at a high temperature which made it challenging to incorporate a high load of CTP (50%) into PAN graphitic structure effectively. It is believed that the balance for CTP loading to contribute in graphitic structure is 25% CTP at high temperature, and higher CTP loading e.g., 50% will result in creating a defect instead of a graphitic structure. In contrast, the thermal condition at a low carbonization temperature is more suitable for high CTP loading to slowly integrate into a graphitic structure. In other words, as CTP contains highly developed polyaromatic rings, a higher intensity of sp^2^ carbon atoms was detected in the U4 sample at low temperatures. However, carbonization at high temperatures showed that the polyaromatic rings are more subjected to create defect carbons instead of generating more graphitic carbons and I_D_/I_G_ ratio of U4 at 1200 °C is slightly higher than the U0 and U2 samples. Alternatively, U4 may require different thermal profiles (such as longer carbonising times but lower temperature) to optimise its graphitisation, which in turn can compromise low-cost carbon fibre concept.

### 3.4. Electrical Resistivity

**Figure 9:**
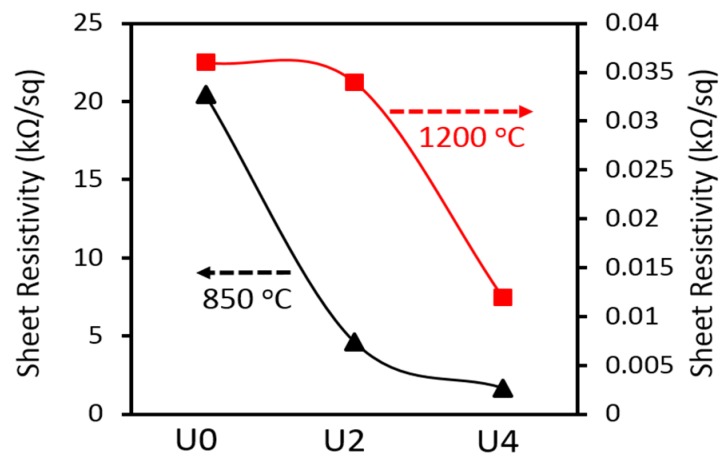
Sheet resistance for samples of U0, U2 and U4 at different carbonisation temperatures.

Pitch-based CFs are often known to have higher electrical and thermal conductivity compared to PAN-based CFs. We have investigated the effect of CTP on electrical resistivity of PAN CFs. The sheet resistivity of the carbonised samples is shown in Figure 9. As seen, electrical resistivity significantly reduced when carbonisation temperature increased from 850 °C to 1200 °C. For example, the electrical resistivity of U0 at 850 °C is about 20 kΩ/sq while this value dropped to 0.036 kΩ/sq at 1200 °C. Such a trend was also observed for the U2 and U4 samples in which the electrical resistivity reduced around 136 and 140 fold, respectively, by increasing the carbonisation temperature. When it comes to the CTP percentage, the electrical resistivity of the carbonised samples at 850 °C reduced significantly from about 20 kΩ/sq for U0 to 4.6 kΩ/sq and 1.6 kΩ/sq for U2 and U4, respectively. In other words, by adding 25% and 50% CTP, the electrical resistivity was decreased by 4 and 12 fold compared to the U0 sample at 850 °C. However, these differences were reduced at 1200 °C to 1.5 and 3 fold in comparison with U0 for the U2 and U4 samples, respectively. It can be also seen that when CTP content in PAN fibres increased from 25% to 50%, a small reduction in electrical resistivity was obtained at 850 °C, suggesting less incremental gains could be made with higher loading.

## 4. Conclusions

The high molecular weight CTP with high carbon content was extracted by heat treatment of a natural coking coal and was used as the precursor materials in the production of low-cost and sustainable CFs. High loadings of CTP were blended with a PAN solution to produce PAN/CTP composite precursors. The rheological investigations showed that although the addition of 25% and 50% CTP increased the viscosity of PAN/CTP solutions, the spinnability was unaffected and fibres with diameters of 0.45 µm and 0.72 µm were produced, respectively. DSC analyses revealed that the addition of 25% CTP into PAN fibres significantly increased the heat release during thermal stabilisation and decreased the activation energy required for thermal conversion. Moreover, the carbon yield obtained by TGA analysis at 600 °C increased by 25% for PAN fibre containing 25% CTP compared to pure PAN fibres. The XRD results showed that the addition of CTP to PAN increases the crystallite size of the fibres. Moreover, a reduction in crystallite size of PAN/50% CTP CFs was obtained while the crystallite size of PAN/25% CTP CFs at carbonisation temperature of 1200 °C remained constant when compared to pure PAN CFs. These results suggest that the inclusion of 25% CTP in PAN could effectively contribute to enhancing the graphitic structure of CFs. Furthermore, Raman analysis demonstrated that the ratio of defect structure to graphitic structure reaches its lowest value when 25% CTP was blended with PAN and carbonised at 1200 °C. Finally, the extremely low electrical resistivity of 4.6 kΩ/sq and 1.6 kΩ/sq were obtained at a moderate carbonisation temperature of 850 °C with addition of 25% and 50% CTP into PAN CFs, respectively. 

## Figures and Tables

**Table 1:** Rheological and structural properties of PAN/CTP solutions.

Sample Code	Viscosity (Pa.s)	Flow Behaviour Index (n)	Diameter (µm)	Electrical Conductivity (mS/cm)
U0	0.768	0.99	0.38 ± 0.13	127.8
U2	0.848	0.95	0.45 ± 0.07	116.4
U4	1.63	0.92	0.72 ± 0.13	104.9

**Table 2:** Thermal characteristics of various samples obtained by DSC and thermogravimetry analyses (TGA).

Sample	*T*_s_ (°C)	Δ*H*_s_ (J/g)	*E*_s_ (kJ/mol)	*T*_c_ (°C)	Carbon Yield at 600 °C (%)
U0	311	1047	145	323	43
U2	316	2106	113	331	54
U4	318	2232	148	334	56

**Table 3:** Brunauer, Emmett and Teller (BET) surface area and pore characteristics of the various CFs.

Carbonisation Temperature	850 °C			1200 °C		
Sample	U0	U2	U4	U0	U2	U4
Specific surface area (m^2^/g)	87.33	110.45	142.86	104.28	134.41	145.12
Pore size (nm)	1.4054	1.6571	3.138	1.5680	1.7491	3.409

**Table 4:** The results extracted from XRD patterns and calculated size of crystallites for precursor, low temperature and high-temperature carbonised samples.

Samples		2ϴ (Degree)	FWHM (Degree)	L_c_ (nm)
**CTP**		20.15	9.36	N/A
**Precursor**	U0	16.83	3.06	2.59
	U2	16.81	3.01	2.63
	U4	16.82	2.90	2.74
**Carbonised @850 °C**	U0	24.43	7.55	1.07
	U2	24.41	7.47	1.07
	U4	24.41	7.51	1.07
**Carbonised @1200 °C**	U0	24.24	7.48	1.07
	U2	24.33	7.49	1.07
	U4	24.05	7.71	1.04
